# Resonance in the Mouse Ventral Tegmental Area Dopaminergic Network Induced by Regular and Poisson Distributed Optogenetic Stimulation *in-vitro*

**DOI:** 10.3389/fncom.2020.00011

**Published:** 2020-02-18

**Authors:** Luuk van der Velden, Martin A. Vinck, Wytse J. Wadman

**Affiliations:** ^1^Swammerdam Institute for Life Sciences, University of Amsterdam, Amsterdam, Netherlands; ^2^Ernst Strüngmann Institute (ESI) for Neuroscience in Cooperation With Max Planck Society, Frankfurt am Main, Germany

**Keywords:** dopamine, network, optogenetics, self-organization, rhythm, resonance, action potentials, multi-electrode array

## Abstract

Neurons in many brain regions exhibit spontaneous, intrinsic rhythmic firing activity. This rhythmic firing activity may determine the way in which these neurons respond to extrinsic synaptic inputs. We hypothesized that neurons should be most responsive to inputs at the frequency of the intrinsic oscillation frequency. We addressed this question in the ventral tegmental area (VTA), a dopaminergic nucleus in the midbrain. VTA neurons have a unique propensity to exhibit spontaneous intrinsic rhythmic activity in the 1–5 Hz frequency range, which persists in the *in-vitro* brain slice, and form a network of weakly coupled oscillators. Here, we combine *in-vitro* simultaneous recording of action potentials from a 60 channel multi-electrode-array with cell-type-specific optogenetic stimulation of the VTA dopamine neurons. We investigated how VTA neurons respond to wide-band stochastic (Poisson input) as well as regular laser pulses. Strong synchrony was induced between the laser input and the spike timing of the neurons, both for regular pulse trains and Poisson pulse trains. For rhythmically pulsed input, the neurons demonstrated resonant behavior with the strongest phase locking at their intrinsic oscillation frequency, but also at half and double the intrinsic oscillation frequency. Stochastic Poisson pulse stimulation provided a more effective stimulation of the entire population, yet we observed resonance at lower frequencies (approximately half the oscillation frequency) than the neurons' intrinsic oscillation frequency. The non-linear filter characteristics of dopamine neurons could allow the VTA to predict precisely timed future rewards based on past sensory inputs, a crucial component of reward prediction error signaling. In addition, these filter characteristics could contribute to a pacemaker role for the VTA in synchronizing activity with other regions like the prefrontal cortex and the hippocampus.

## 1. Introduction

The ventral tegmental area (VTA) is a dopamine nucleus in the midbrain, alongside the substantia nigra and the red nucleus. VTA dopamine neurons exhibit spontaneous low-frequency rhythmic spike activity (Werkman et al., 2001; Bayer et al., 2007). The rhythm is intrinsic and is even retained in acute slices and in isolated VTA dopamine neurons (Koyama et al., 2005). Experimental research and modeling studies point to at least two distinct mechanisms that underlie this oscillatory activity (Khaliq and Bean, 2010; Drion et al., 2011): it is either based on sub-threshold calcium oscillations or on the presence of the persistent sodium current. VTA dopamine neurons have direct synaptic connections between each other but they also connect through a local interneuron network (Bayer and Pickel, 1990; Omelchenko and Sesack, 2009). In addition they are likely to interact through dopamine volume transmission (Cragg et al., 2001). This phenomenon implies that the firing neurons create an oscillating extracellular dopamine concentration that results in auto inhibition of the population and helps to synchronize all neurons (van der Velden et al., 2017). The VTA network can therefore be considered as a network of weakly coupled oscillators.

Oscillatory rhythms in the brain create the context for encoding information, specifically in the detailed timing of their firing (Buzsaki, 2009). The VTA is implicated in a low frequency local field oscillation (with spectral energy focused in the 1–5 Hz band) which synchronizes it with the prefrontal cortex and the hippocampus during memory processing (Battaglia and McNaughton, 2011; Fujisawa and Buzsáki, 2011). An additional hypothesis states that VTA pacemaker activity entrains the prefrontal cortex and the hippocampus (Fujisawa and Buzsáki, 2011). The timing information in its output plays an important role in stimulus-reward processing (Schultz, 1997) especially where activities need to be related at relatively long time scales.

Elucidating the population dynamics of the VTA dopamine neurons requires simultaneous recording of the spiking activity of as many neurons as possible, which can be accomplished in acute brain slices positioned on a multi-electrode-array (MEA). The slice is void of external input, which restricts the preparation, but also prevents complications from uncontrolled external input and thus simplifies the interpretation (Berretta et al., 2010; van der Velden et al., 2017). Specific activation of neurons at a very fast time scale (ms) can be accomplished with optogenetics. Crossing the floxed ChR2-EYFP mouse (Madisen et al., 2012) with the Pitx3 Cre driver mouse (Smidt et al., 2012) expresses light sensitive excitatory channelrhodopsin specifically in dopamine neurons. We can then use pulsed laser driven activation for precise manipulation of spike timing in individual neurons as well as at the level of the entire population (Deisseroth, 2010; Fenno et al., 2011) during long-term recordings without noticeable photo-toxicity (Cardin, 2012).

This study focuses on the lateral VTA, which mainly contains mesolimbic projecting dopamine neurons with a classic dopamine neuron phenotype (Lammel et al., 2008). The auto-oscillatory VTA dopamine neurons (van der Velden et al., 2017) and in particular their response to rhythmic and stochastic input were recorded. Their resonance characteristics were probed at the population level using regular (fixed frequency) as well as Poisson distributed (noisy) pulsed input. Driving populations of weakly coupled oscillators with such stimulation regimes has theoretically been worked out and reveals interesting dynamics (Hata et al., 2010). Experimental and modeling research showed that populations of oscillators can encode common input into their oscillatory output, even when the input is noisy (Ermentrout et al., 2008). We demonstrate here that the resonance characteristics of the VTA dopamine neuron population in response to temporally patterned stimulation reveal emergent properties at the network level.

## 2. Methods

### 2.1. Experimental Animals

Adult male and female mice between 6 and 11 weeks old were used in the experiments. They were housed in a 12/12 light dark cycle and received water and food ad libitum. All procedures and methods were approved by the ethical committee for animal experimentation of the University of Amsterdam.

### 2.2. Optogenetic Expression

The Pitx3 Cre-driver mouse (Smidt et al., 2012) was crossed with the LoxP-ChR2-EYFP mouse (Madisen et al., 2012) to express channelrhodopsin-2 (ChR2) in the midbrain dopamine neurons. Both mice had a black-6 genetic background and were homozygous for their respective allele of interest. First generation offspring, heterozygous for both alleles (male and female), were used in the experiments. The Cre expression in the Pitx3-Cre mouse completely overlapped with the tyrosine hydroxylase expressing dopamine neurons in the lateral VTA (Smidt et al., 2012; Luk et al., 2013). In our F1 offspring we checked the presence of the two proteins in the lateral VTA dopamine neurons using fluorescent immuno-staining with GFP and Pitx3 anti-bodies, employing confocal and wide field imaging (see below).

### 2.3. Immunocytochemistry

**Figure 1:**
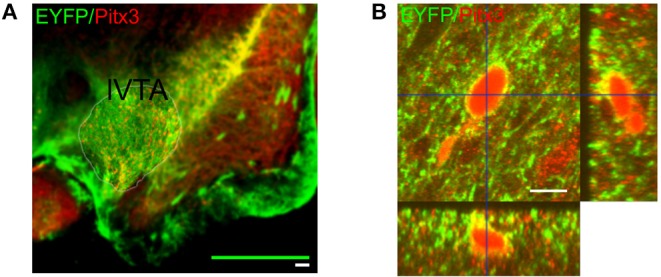
Expression of the excitatory channelrhodpsin-2 channel in VTA dopaminergic neurons. **(A)** Representative wide-field micrograph showing the expression pattern of both EYFP (green) and Pitx3 (red) throughout the midbrain. One lateral side of a coronal midbrain slice is shown (lateral VTA). The midline is to the left side of the figure. The white scale bar corresponds to 100μm and the green bar indicates the outer size of the square MEA recording device that was optimally placed over the indicated VTA region (thin yellow contourline). The laser illumination spot fell well inside the MEA recording area as could be easily judged from the photo-electric artifact that it induced into the MEA leads. **(B)** Representative orthogonal projected confocal micrograph showing a EYFP+/Pitx3+ neuron located in the lateral VTA. Co-localization of Pitx3 expression (red) and ChR2-EYFP (green) is seen as a yellow coloring of the neuronal membrane. The orthogonal side views further illustrate the membrane expression of ChR2-EYFP. The scale bar corresponds to 10 μm.

Free floating acute brain slices (100 μm), were fixed for 1 h at room temperature with 4 % paraformaldehyde in phosphate buffered saline (PBS) pH 7.4. Subsequently washed with PBS and blocked for 4 h with 10 % normal goat serum plus 0.25 % Triton X-100 in PBS at room temperature. The slices were incubated overnight with the primary antibodies GFP (1:750; Abcam; ab13970) and Pitx3 [1:1000; Smidt et al. (2000)] diluted in 3 % normal goat serum plus 0.25 % Triton X-100 in PBS at 4°C. The following day, the slices were washed with PBS and incubated for 4 h with the secondary antibodies Goat anti-Rabbit Alexa Fluor 555 (1:1000; Molecular Probes; A-21428) and Goat anti-Chicken Alexa Fluor 488 (1:1000; Molecular Probes; A-11039) diluted in 1 % normal goat serum plus 0.025 % Triton in PBS at room temperature. Sections were washed with PBS and cover slipped with Vectashield including DAPI (Vector Laboratories, Burlingame, CA, USA). Both ChR2-EYFP and Pitx3 were expressed within dopamine nuclei in our midbrain preparation (Figure 1A). Confocal images showed Pitx3 expression within the soma (red) combined with ChR2-EYFP expression (green) on the membrane, which lead to yellow coloring (Figure 1B). To characterize the co-expression, Z-stack images were acquired using a Zeiss LSM510 confocal laser scanning microscope fitted with a 63x objective. The orthogonal views enhanced the view of the co-localized expression (Figure 1B).

### 2.4. Slice Preparation and Electrophysiology

Mice were decapitated, the midbrain was dissected and kept at 4°C in artificial cerebral spinal fluid (ACSF) containing (in mm) NaCl 120, KCl 3.5, CaCl_2_ 2.5, NaH_2_PO_4_ 1.25, MgSO_4_ 1.3, NaHCO_3_ 25, D-glucose 10 which was bubbled with carbogen (95 % O_2_; 5 % CO_2_) to set pH at 7.4. Coronal slices were cut 300 μm thick from caudal to rostral using a VT1000S vibratome (Leica, Wetzlar, Germany). The fading of the substantia nigra during progressive slicing was a marker for the caudal-medial part of the VTA. One or two slices containing the caudal and medial part of the VTA were used for the experiments. Slices were incubated for 30 min at 32°C directly after slicing and were kept at room temperature until the start of the experiment. During recording in a MEA-1600 multi-electrode system (MultiChannel Systems, Reutlingen, Germany) the slice was kept at 32°C and continuously perfused with ACSF bubbled with carbogen. The VTA was identified in the mid-brain slice and positioned on top of the 3D MEA (Qwane Biosciences, Lausanne, Switzerland) containing 60 protruding electrodes (8*8 square layout) of 30 μm diameter and 100 μm spacing in order to record the spontaneous activity of multiple single-units (Olivier et al., 2002; van der Velden et al., 2017, 2019). All chemicals were obtained from Sigma-Aldrich (Zwijndrecht, the Netherlands).

### 2.5. Data Acquisition

**Figure 2:**
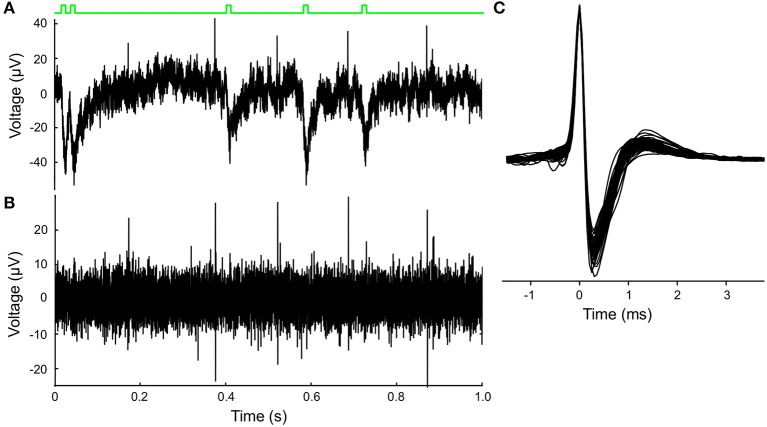
Extracellular MEA recordings and optogenetic stimulation of VTA dopamine neuron activity. **(A)** Laser stimulation and unit recording illustrated in a 1s excerpt from a 720 s recording. Raw extracellular recording from one of the 60 MEA electrodes showing positive spikes that were clearly exceeding the noise level, and the negative photo-electrical artifacts initiated by the laser pulses. Schematic green line at the top depicts the timing of laser pulse stimuli (Poisson process). **(B)** Result of high-pass (>225 Hz) filtering of the signal in **(A)** which emphasizes the neuronal spikes and suppresses the photo-electrical artifact. **(C)** Superimposed spike-waveforms (cutout), which were averaged and normalized to the peak amplitude for each recorded VTA dopamine neuron (*n* = 73). Waveforms show the classic tri-phasic waveform of midbrain dopamine neurons.

The MEA recordings showed identifiable spikes, the extracellular representation of local action potentials, of 30–130 μV amplitude superimposed on a background noise of ~15 μV (Figure 2A). The raw signal was high pass filtered at 225 Hz using a second order Butterworth filter and then sampled at 20 kHz (Figure 2B). Positive voltage peaks were detected, with a relatively low threshold in order not to miss spikes. Negative polarity spikes were rarely recorded and less suitable for analysis due to the negative polarity photoelectric artifact of the laser pulse stimulation on the MEA electrode leads (Figure 2A, green trace at the top indicates laser activation). Patches of signal containing the peak waveforms 3 ms around the peak were collected. K-means clustering was used to define the largest two principle components and the peak amplitudes of the waveforms. The auto-correlation and inter-spike-interval distribution of the peaks in the clusters were examined to identify clusters consisting of neuronal spikes. For electrodes that contained more than one neuron the most reliably recorded neuron was selected, most often based on peak amplitude (van der Velden et al., 2017).

### 2.6. Laser Stimulation Protocols

A 532 nm GL532T3 laser (Shanghai Laser & Optics Century Co., Shanghai, China), with an output of 320 mW, was coupled to a glass fiber, with 100 μm core radius and 0.22 NA, via a HPUC-23AF optic coupler (OZ optics, Ontario, Canada). Laser pulse stimulation to the superfused brain slice was applied from above. The fiber made contact with the perfusion liquid, but did not touch the slice. The distance between the MEA and the laser was ~3 mm. The light intensity at the recording sites in this configuration had a lower bound of 5 mW/mm^2^ (Deisseroth, Stanford). The calculation is based on light traveling through brain tissue only, but we also had travel distance through water. With this light intensity we do not expect to highly excite the neurons, but it is within the range reported in the literature (Madisen et al., 2012). The laser light induced a negative photoelectric artifact on the MEA electrodes so that visual inspection of the decay of this effect allowed us to ensure that the effective laser light spot was limited to the recording area of the MEA and covered the majority of the relevant electrode contacts. A Raspberry-Pi micro-controller (Raspberry-Pi foundation, UK) was programmed in Python to digitally control the timing of the laser stimuli (pulse duration always 10 ms) with a time resolution of around 20 μs. Two fundamentally differently timed stimulation protocols were used: The first one consisted of six segments of 240 s periods of laser stimulation (total 1440 s stimulation) interleaved with six segments of 240 s of no laser stimulation. In the on-period the laser either fired at a fixed 2 Hz rate or it produced a Poisson distributed stimulus train (exp(-λt)). The expectation density of the Poisson stimulation was 5 events/s and had its dominant power was distributed over the 1–5 Hz frequency range. The second protocol consisted of six 30 s periods of laser stimuli for each frequency (180 s per frequency). The laser stimulation frequency took fixed values between 0.5 and 5.5 Hz in 0.5 Hz steps. These protocols were applied in random order (repeated six times each).

### 2.7. Spike Train Analysis

**Figure 3:**
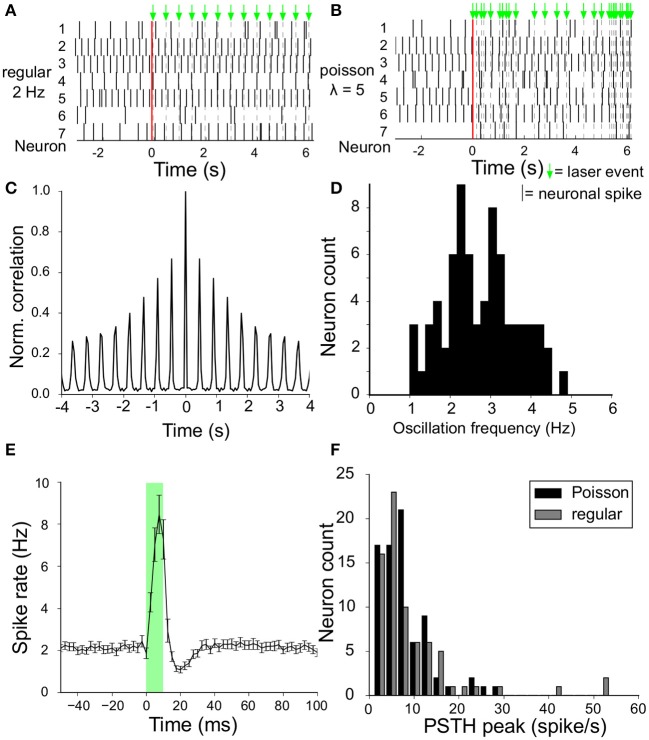
Spontaneous activity and modulation by laser stimuli. **(A)** Raster plot of simultaneously recorded spike activity of a sample of seven VTA dopamine neurons in the same slice, before and during laser stimulation (onset at *t* = 0, vertical red line). Laser stimuli were delivered at a frequency of (2Hz), and are depicted by the green arrows above the top panel. **(B)** Same as **(A)** but now with laser pulses with a Poisson interval distribution (having an expectation value of 5 events/s). **(C)** Auto-correlation of the spontaneous activity of an example VTA dopamine neuron. The oscillation frequency was derived from the side-lobes in the auto-correlation. **(D)** Distribution of oscillation frequencies of all recorded VTA dopamine neurons (*n* = 73) from 7 experiments (=slices/animals). **(E)** Instantaneous firing rate around the laser stimulus, calculated from the Peri-Stimulus-Time-Histogram (2.5ms bin size) accumulated from all neurons (*n* = 73) for the 2Hz regular stimulation protocol. A sharp peak is followed by a trough in the spike rate during and after the laser stimulus given at (*t* = 0, transparent green) with stimulus duration of 10ms). **(F)** The maximum increase in instantaneous firing rate (peak minus baseline value) of the curve in **(E)** for all neurons is aggregated into a histogram to show the distribution of laser stimulation efficacy (*n* = 73). Results shown for both regular (gray) and Poisson stimulation (black).

The instantaneous firing rate (with 2.5 ms resolution) around the laser stimulus (at *t* = 0) was calculated from the Peri-Stimulus-Time-Histogram (PSTH) averaged over all recorded neurons (Figure 3E). Spike rate is expressed in spikes/s or Hz. The same graph was constructed for each neuron and we defined the efficacy of the laser stimulation for that neuron by the induced increase in firing rate above baseline level.

Rhythmic firing of VTA dopamine neurons can also be demonstrated as peaks in their auto-correlation function (Figure 3C). The oscillation frequency quantifies the preferred intrinsic rhythm of the neuron and was calculated as the mean interval between the side-lobes in the auto-correlation function, including the interval between the zero-lag peak and the first side lobe.

The irregularity of neuronal firing in the VTA was assessed using a measure of local variation, which quantifies the similarity between consecutive inter-spike-intervals (ISI), as in van der Velden et al. (2019). The local variation (LV, Shinomoto et al., 2005, 2009) of a spike train ranges from 0 (perfectly regular firing) to 1 (Poisson distributed firing) and above 1 for burst-like firing and is given by:

(1) $$\begin{array}{r} LV=\frac{3}{n-1}\overset{n-1}{\underset{i=1}{\sum }}\left(\frac{T_{i}-T_{i+1}}{T_{i}+T_{i+1}}\right) \end{array}$$

where *T*_*i*_ is the i-th interval in the spike train that contains n spikes.

The Phase-Response-Curve (PRC) measures the laser-pulse-induced perturbation of a neurons' spike cycle. The phase of the spike cycle was normalized between zero and one, taking the oscillation frequency as a reference. A laser pulse could either delay the time to the next spike (negative phase shift) or advance it (positive phase shift). Averaging the PRC across neurons was done by binning the stimulus phase. The strength of the synchrony between a neuron's spike train and the laser pulses was quantified with the Pairwise-Phase-Consistency (PPC), as previously defined (Vinck et al., 2010) and validated (van der Velden et al., 2019). The PPC estimates the similarity of the relative phases of the point processes with respect to a chosen reference frequency and estimates the square of the classic Phase-Lock-Value (Lachaux et al., 1999). The PPC is an unbiased metric of phase-synchronization that scales with the square rather than the square root of the coherence and phase locking value (Vinck et al., 2010). To compute the PPC, spike and stimulation pulse trains were binned at 1 ms bins and a (Hanning) windowed Fourier Transform was computed on a series of time segments of the spike train. The length of the time segments was set to contain a fixed number of cycles of the reference frequency of interest (e.g., 5 cycles). The relative phase is defined as the complex argument of the classic spectral coherence (Lachaux et al., 1999; Vinck et al., 2010). From these relative phases the PPC was computed:

(2) $$\begin{array}{r} PPC=\frac{2}{N\left(N-1\right)}\overset{N-1}{\underset{j=1}{\sum }}\overset{N}{\underset{k=j+1}{\sum }}cos\left(\theta_{j}-\theta_{k}\right) \end{array}$$

where there are *N* time segments, segment *j* has relative spike phase θ_*j*_ and segment *k* has relative spike phase θ_*k*_, computed in respect to a chosen reference frequency. For the interleaved 2 Hz and the Poisson protocol we computed the PPC at selected frequencies independent of the laser pulse frequency. In the stepped frequency protocol we computed the PPC only at the frequencies of the laser stimuli. The PPC was also used to assess the synchrony between spike trains simultaneously recorded from pairs of neurons. To disentangle the synchrony between neurons driven by common laser input and the part due to direct interactions between the neurons, we computed the partial coherence (Rosenberg et al., 1989, 1998; Brett et al., 2009) as defined in Sun et al. (2004); Brett et al. (2009). The spectral estimation for the coherence computation was performed using Welch's algorithm with overlapping windows of 5 s (Welch, 1967; Brett et al., 2009).

### 2.8. Experimental Design and Statistical Analysis

Independent experiments were performed on 7 slices during which in total 73 neurons were recorded, each slice was obtained from a different animal. Differences between experiments (=slices/animals) were tested with an ANOVA on two indicator parameters: the neuronal oscillation frequency and the laser induced spike probability. Detailed statistics were performed across all recorded neurons with *n* = 73 degrees of freedom. Exact *p*-values are given in the results section. The main statistical test used across neurons is the Spearman's rank correlation between the parameter of interest and the oscillation frequency of the neurons. Unless otherwise mentioned, all values reported in this study are given as mean and standard error of the mean (mean ± sem). In graphs the shaded area around a line indicates ± sem. Unless otherwise mentioned, direct comparison of two means was performed with Student's t-test, after checking for normality. Comparison of multiple groups was performed with ANOVA and *post-hoc* testing. The variance of the PPC for individual neurons was estimated with a jackknife method for each frequency. The variable *n* indicates the number of observations, usually the number of neurons *n* = 73; *p* < 0.05 is assumed to reject the null hypothesis. Numpy/Scipy (Oliphant, 2007), Pandas (McKinney, 2010) and NIPY (Brett et al., 2009) packages for the Python programming language were used to perform the analysis. Data visualization was performed with Matplotlib (Hunter, 2007).

## 3. Results

### 3.1. Spontaneous Activity Modulation

VTA dopamine neurons spontaneously fire rhythmic action potentials. A raw (Figure 2A) and filtered (Figure 2B) trace recorded from a single MEA electrode illustrate the activity of a neuron and the laser-pulse induced artifacts (photoelectric effect observed in the MEA leads). In the high pass filtered signal the neuronal spikes are easily detected above the (high-frequency) noise and the artifacts can be suppressed. Neuronal firing activity is also present in the absence of the laser stimulation, as reported previously (van der Velden et al., 2017, 2019). We calculated the amplitude-normalized averaged spike-waveform for each individual neuron and superimposed them in Figure 2C to demonstrate the highly characteristic classic tri-phasic waveform of midbrain dopamine neurons (Werkman et al., 2001; van der Velden et al., 2017, 2019). All neurons displayed the classic tri-phasic waveform of midbrain dopamine neurons (Werkman et al., 2001; van der Velden et al., 2017, 2019). The intrinsic oscillation frequency in the spike activity was computed from the time interval between peaks in the auto-correlations of the individual neurons as in van der Velden et al. (2019) (example in Figure 3C). Figure 3D shows the distribution of the intrinsic oscillation frequencies of all the neurons (mean ± standard deviation:2.9 ± 0.9. The oscillation frequencies varied between 1 and 5 Hz, which matches well with the Local Field Potential power spectra of the VTA *in vivo* Fujisawa and Buzsáki (2011). This variation in oscillation frequency was observed between the neurons in the same slice and did not differ between experiments (=slices) (ANOVA, *n* = 7, *p* = 0.56). In previous work we have shown that the spontaneous activity of the lateral dopamine neurons is highly rhythmical and variation in regularity did not lead to separable neuronal populations (van der Velden et al., 2019). The raster plots in Figures 3A,B show 8 s spike trains (excerpts out of the six recording periods of 240 s) simultaneously recorded from seven VTA dopamine neurons in the same slice under regular 2 Hz laser stimulation (Figure 3A) and Poisson distributed laser stimulation (λ = 5, Figure 3B). The average PSTH of all neurons (*n* = 73) during 2 Hz regular stimulation (Figure 3E) showed a strong increase in spike rate after the onset of the laser pulse, immediately followed by a trough. Nonetheless, modulation by the laser stimulation was not equally effective for all neurons and showed considerable variation (Figure 3F). This shows that the optogenetic stimulation was, as expected from the expression patterns of ChR2 (Figure 1), strongly enhancing neuronal firing rates directly after the laser pulse onset.

### 3.2. Phase Response and Optogenetic Stimulation Strength

**Figure 4:**
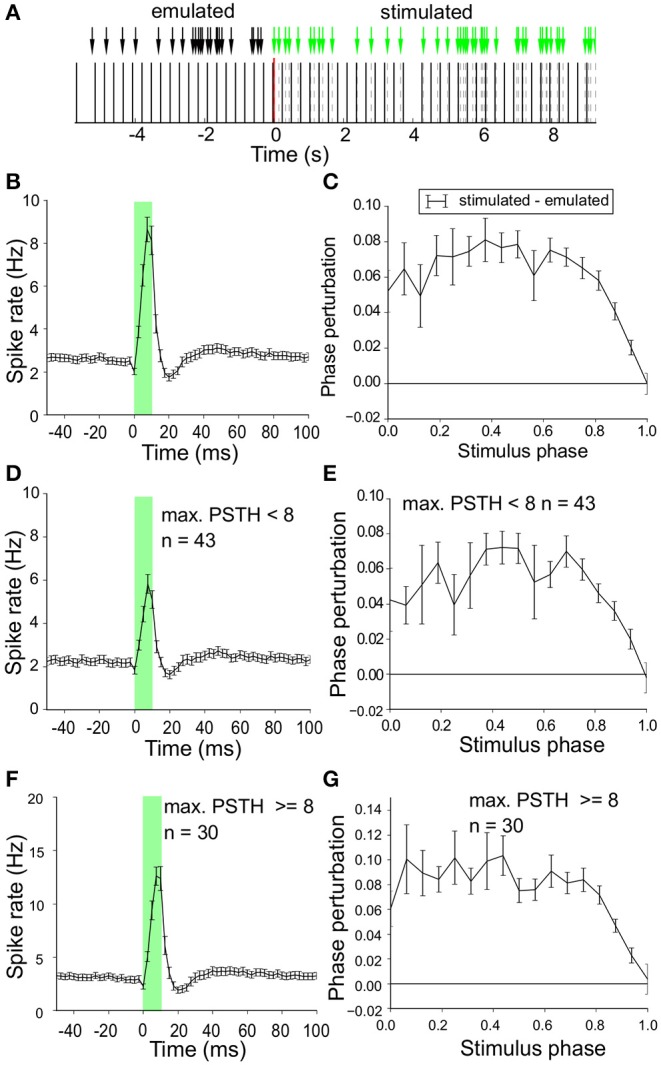
Phase shift induced by laser stimulation. **(A)** Poisson stimulation protocol (λ = 5) during a stimulus off to on transition at *t* = 0. In the off condition we recorded baseline neuronal activity and for our analysis we emulated surrogate laser pulses (black arrows) with exactly the same time pattern but zero intensity. This allows us to perform identical assessment of the phase shift during laser on and off (control). **(B)** Mean instantaneous firing rate (spike/s) around the laser pulse (details as in Figure 3E) for all neurons in response to Poisson stimulation with a sharp peak during laser stimulation (green overlay) followed by a trough. **(C)** Phase-Response-Curve (PRC) (mean ± sem) for all neurons in response to Poisson stimulation. The PRC for the surrogate laser stimulation was subtracted to correct for potential bias.The x-axis represents the cycle duration, which is proportional to the neurons' intrinsic oscillation frequency. Spike phase advance (positive values) was largest around halfway the spikes (phase ~ 0.5). **(D)** Mean instantaneous firing rate (spike/s) around the laser pulse (details as in Figure 3E) for neurons with a relatively low optogenetic efficacy (relative amplitude of peak above baseline < 8 spike/s). A sharp peak is followed by a trough, similar to the population result in **(B)**. **(E)** PRC [similar to **(C)**] of neurons with low optogenetic efficacy (relative amplitude of peak above baseline < 8 spike/s). The phase advance due to laser stimulation is largest halfway the spikes (phase ~ 0.5). **(F)** Mean instantaneous firing rate (spike/s) around the laser pulse (details as in Figure 3E) for neurons with high optogenetic efficacy (relative amplitude of peak above baseline ≥ 8 spike/s). A sharp peak is seen followed by a trough, similar to the population result in **(B)**. **(G)** PRC [similar to **(C)**] of neurons with strong optogenetic efficacy (PSTH peak ≥ 8 spike/s).

The spike time modulation by the optogenetic pulses was first studied during Poisson laser stimulation (λ = 5) with Phase-Response-Curves (PRCs). Phase was normalized to values between 0 and 1 using the neuron's intrinsic oscillation frequency. The phase-shift of the next spike induced by the laser stimulus was determined as a function of the phase of the stimulus (Figure 4). To control for possible random bias in our procedure we ran an identical PRC analysis on emulated laser pulses (i.e., on a surrogate laser pulse train with exactly the same timing, but no laser activation) during interleaved baseline recordings (black arrows in Figure 4A). The PRC for the surrogate train was then subtracted from the PRC of the actual laser train. The PRC, averaged over all neurons, showed an ~8 % phase advance due to laser stimulation (Figure 4C). The maximal phase perturbation was observed halfway the spike cycle (phase 0.5), but this peak was not very pronounced. The mean instantaneous spike rate of this group of neurons around the stimulus is illustrated in Figure 4B and in essence hardly different from the one in Figure 3E for regular stimulation.

The shape of the PRC could well be related to the efficacy of the laser stimulation as measured in Figure 4B. Therefore we split the recorded neuron population based on their stimulation efficacy around the mean (8 spikes/s, Figure 3F) and obtained two groups (efficacy < 8Hz, *n* = 43, efficacy ≥ 8Hz, *n* = 30). Figures 4D,F show the instantaneous spike rate around the laser pulse averaged for each group of neurons. The shape of the curves is similar with a sharp peak during laser stimulation (green overlay) followed by a trough in spike rate. The PRCs, mean over all neurons in the group, are given in Figures 4E,G and both show phase-advancing induced by the laser reaching its maximal value at a stimulation phase halfway the spike cycle (around phase 0.5). The phase advance induced by the laser is slightly larger in the high efficacy group but the two PRCs did not show qualitative differences in the timing response to optogenetic pulse stimulation. These findings indicate that laser stimulation directly after a neuron has fired (start of the cycle) or directly before a neuron is expected to fire (end of the intrinsic oscillation cycle) have a relatively small effect on the timing of the next spike, but laser stimulation in the middle of the cycle can induce up to 10 % phase advance in the timing of the next spike. Similar results were obtained with regular 2 Hz stimulation (not shown), but Poisson stimulation sampled the phase relations between neuronal spikes and laser pulses more effectively, without the incidental risk of very strong phase locking.

### 3.3. Resonance to Pulse Stimulation Under Two Stimulus Regimes

**Figure 5:**
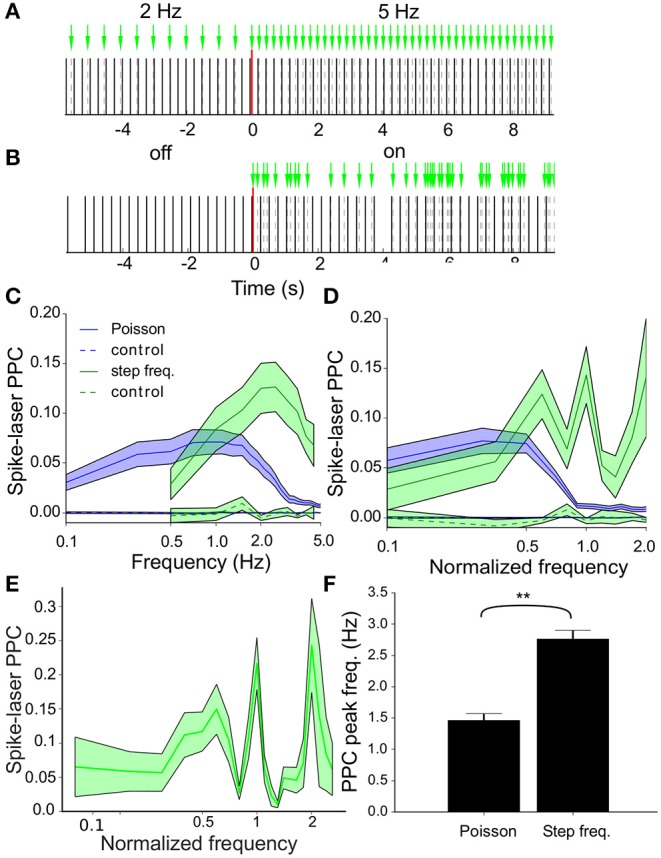
Resonance of the VTA dopamine neuron population. **(A)** Step frequency protocol with frequency transition between two 30s recording periods transitioning from 2Hz to 5Hz stimulation. In addition to the actual protocol a control analysis was performed on an emulated (surrogate) stimulation protocol applied to baseline recordings of the neurons. **(B)** Laser stimulation as a Poisson process with 5 events/s (λ = 5) with laser-off and laser-on periods interleaved. **(C)** Pairwise phase consistency (PPC) spectra averaged across all neurons (*n* = 73) during stimulation [regular (green) and Poisson (blue), (mean ± sem)] measuring the phase synchronization between neuronal spikes and laser pulses. During regular stimulation a peak in resonance between neurons and laser was seen around 2Hz, whereas the highest resonance was observed around 1Hz during Poisson stimulation. The controls (dashed lines in the same color scheme) show that the correlation was not due to biasing factors such as incidental synchrony between laser and neuron. **(D)** PPC spectra averaged across all neurons (*n* = 73) during stimulation [regular (green) and Poisson (blue), (mean ± sem)]. Before averaging, the frequency values (x-axis) were normalized by dividing by the oscillation frequency for each individual neuron. During regular stimulation, we observed speaks in resonance around the oscillation frequency (corresponding to frequency value of 1.0) and its harmonics. Poisson stimulation led to resonance at relatively lower frequencies as compared to stimulation with regular pulse trains. **(E)** Same as **(D)** but now at a higher resolution, only for regular stimulation. **(F)** Bar plots of the peak spike-laser PPC frequency. The peak resonance-frequency is higher during step frequency (i.e., regular) stimulation as compared to Poisson stimulation. By contrast, the oscillation frequency (not shown) did not change between protocols.

The influence on the spike timing quantified by the PRCs in Figure 4 is quite moderate, but these effects can have a cumulative effect on the synchrony between neuronal spikes and laser pulses. We used the Pairwise-Phase-Consistency (PPC, Vinck et al., 2010) to quantify this synchrony between spiking and laser (spike-laser PPC) and studied resonance at various frequencies. Two experimental laser stimulation protocols were used. The step frequency protocol sampled regular stimulation frequencies between 0.5 and 5.5 Hz with 0.5 Hz resolution (Figure 5A). Each frequency was applied six times for 30 s and for each neuron the spike-laser PPC was computed between neuron and laser at these frequencies. The Poisson protocol consistent of randomly timed pulses during 6 periods of 240 s interleaved with as many periods of baseline recordings. As controls for both protocols, identical spike-laser PPC analyses were performed between emulated laser pulses (i.e., a surrogate laser stimulation train) and baseline firing activity.

Spike-laser PPC spectra were obtained for all recorded neurons during both protocols and averaged across neurons (Poisson: blue and regular: green, Figure 5C). Strong resonance between the neurons and laser was found for both protocols, but interestingly at different dominant frequencies, namely at around 2 Hz for the step frequency stimulation and around 1 Hz for Poisson stimulation. By contrast, the surrogate laser controls for both protocols showed PPC values very close or indistinguishable from zero at all frequencies. The relation between these spike-laser synchrony curves and the intrinsic oscillation frequency (mean ± sd: 2.9 ± 0.9, Figure 3D) was further studied. To this end, the spike-laser PPC spectrum frequency axis of each neuron was divided by its oscillation frequency, before averaging the PPC spectra across neurons (Figures 5D,E; D at lower resolution than E). For regular stimulation we observed peaks at the oscillation frequency (1.0) and peaks at integer fractions or multiples of the intrinsic oscillation frequency at 0.5 and 2.0 times (Figures 5D,E). Poisson stimulation shows a different pattern where resonance between laser and neuron decreases above the even sub-harmonic of the oscillation frequency (0.5) (Figures 5D,E). The oscillation frequencies did not differ between the baseline measurements of both protocols (delta osc. freq.: − 0.013 ± 0.08, t-test: *p* = 0.87, *n* = 73), while the peak spike-laser PPC frequency increased from Poisson to step frequency (delta PPC peak freq.: 1.3 ± 0.18, t-test: *p* < 0.001, *n* = 73) (Figure 5F).

**Figure 6:**
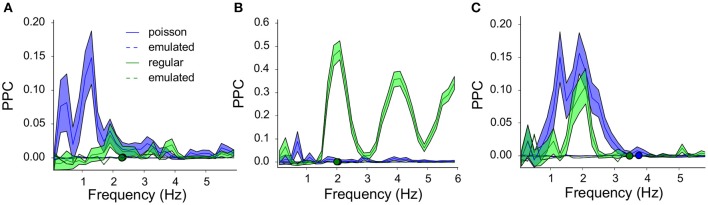
Three example neurons and their resonance spectra illustrate non-linear, band-pass filtering by the VTA dopamine neurons: The response to all frequencies (Poisson stimulation) is not a simple sum of the response to specific frequencies (regular stimulation). **(A)** PPC spectra for a neuron oscillating at around 2.3Hz (green and blue overlapping circles), during regular (green, 2Hz) and Poisson stimulation [blue, λ = 5, (mean ± sem)]. The average of the 100 emulated (surrogate) controls showed PPC values very close to zero. Regular stimulation showed some structure around 2Hz, but synchrony between neuron and laser was stronger for the Poisson stimulation. A visible harmonic structure is related to the neurons' oscillation frequency (2.3Hz) and the laser frequency (2Hz). **(B)** As in **(A)** for a neuron oscillating at 2Hz. During regular stimulation (green) strong synchrony was seen at 2Hz and its even harmonics (4, 6Hz), which were absent during Poisson stimulation. **(C)** As in **(A)** for a neuron oscillating around 3.5–4Hz (green and blue circles). During both protocols resonance was seen at 2Hz).

Example PPC spectra for three neurons are shown in Figure 6. The intrinsic oscillation frequencies are plotted as circles for the two protocols (Poisson blue and regular 2 Hz green). The PPC spectra for the controls (averaged over 100 randomized runs of surrogate laser runs) are shown as dashed lines in the same color scheme. The three neurons show different resonance behaviors; Figure 6A shows resonance to low frequencies during Poisson stimulation; Figure 6B shows high resonance at and above its oscillation frequency during regular stimulation and Figure 6C shows resonance for both protocols below its oscillation frequency. The oscillation frequency of the neuron shown in Figure 6B coincides with the laser stimulation frequency (1:1), which leads to strong phase locking. The other two neurons could contain other modi and beat tones (based on the difference between the laser pulse and oscillation frequency), which leads to these more elaborate spectra.

**Figure 7:**
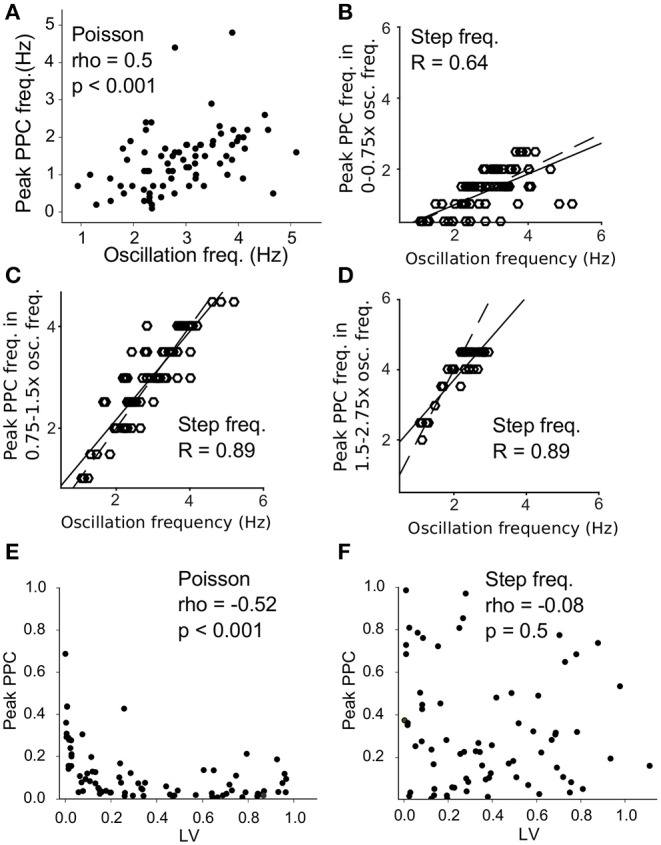
Resonance at different ratios of the intrinsic oscillation frequency for Poisson and regular stimulation. **(A)** Intrinsic oscillation frequency (x-axis) vs. the peak frequency of spike-laser coupling (PPC) for Poisson stimulation. **(B)** Intrinsic oscillation frequency (x-axis) vs. the peak frequency of spike-laser coupling (PPC) for regular stimulation, computed in the frequency range of 0-0.75x the intrinsic oscillation frequency. The dashed line shows the 1:2 equality line, and the solid line the regression fit (R value computed with Pearson). **(C)** Same as **(B)** but now computed for the 0.75–1.5x the intrinsic oscillation frequency range. **(D)** Same as **(B,C)** but now computed for 1.5–2.75x the intrinsic oscillation frequency range. Note that for neurons with a high intrinsic oscillation frequency range, we likely underestimated the peak frequency of spike-laser coupling because of the range of laser stimulation. **(E)** Local Variance (LV) vs. peak PPC during Poisson stimulation. Highly regular firing neurons resonated stronger to the laser pulses as seen by a higher peak PPC. **(F)** LV vs. peak PPC during step frequency stimulation. High resonance to the laser stimulation (high peak PPC) was not correlated to the intrinsic firing regularity of the neurons.

To further quantify the difference in resonance between the two protocols, we measured the strongest resonance frequency as the frequency with the highest spike-laser PPC value between neuronal spikes and laser pulses (peak PPC frequency) for each neuron. The oscillation frequency of the neuron correlated with the peak spike-laser PPC frequency (spearmanρ = 0.5, *p* < 0.001, Figure 7A) for the Poisson protocol, with maximum spike-laser PPC values around 50% of the intrinsic oscillation frequency. For the regular stimulation protocol, we computed correlations between intrinsic oscillation frequency for three different ranges separately, because neurons showed three resonant peaks at 0.5, 1, and 2 times the oscillation frequency (Figure 5E). These ranges were 0–0.75 the intrinsic oscillation frequency, 0.75–1.5 the intrinsic oscillation frequency, and 1.5–2.75 times the intrinsic oscillation frequency. We found that in these three ranges, there was a very strong correlation between the intrinsic oscillation frequency and the peak frequency of spike-LFP PPC. In the 0–0.75 range (times the oscillation frequency), we observed that neurons showed maximum spike-laser PPC values around 0.5 the oscillation frequency, and that neurons with a higher intrinsic oscillation frequency also had a higher peak spike-laser PPC frequency (*R* = 0.64, *p* < 0.001) (Figure 7B). In the 0.75–1.5 range (times the oscillation frequency), we observed that neurons showed maximum spike-laser PPC values around 1 times the oscillation frequency, with again a strong positive relationship between intrinsic oscillation frequency and peak spike-laser PPC frequency (*R* = 0.89, *p* < 0.001) (Figure 7C). In the 1.5–2.75 range (times the oscillation frequency), we observed that neurons showed maximum spike-laser PPC values around 2 times the oscillation frequency, with again a strong positive relationship between intrinsic oscillation frequency and peak spike-laser PPC frequency (*R* = 0.89, *p* < 0.001) (Figure 7D). Note that because we stimulated with regular laser pulses only up to 4.5 Hz, we likely underestimated the peak spike-laser PPC frequency for neurons with the highest intrinsic oscillation frequency, which causes a departure from the 1:2 trend for neurons with the highest oscillation frequency.

We further wondered how the electrophysiological properties of neurons during baseline (i.e., outside laser stimulation periods) correlated with entrainment by the laser. We found that during Poisson stimulation, the most highly regular firing neurons (low LV, as measured during baseline) showed the strongest resonance (high peak PPC, Figure 7E). This relation was not seen for stepped frequency stimulation; high PPC values did not correlate with high regularity (Figure 7F). To conclude, we found that VTA dopamine neurons show resonant filtering of extrinsic inputs around 0.5, 1, and 2x their intrinsic oscillation frequency when these extrinsic (laser) inputs where regular, but responded more strongly to a 0.5x sub harmonic of their oscillation frequency when the extrinsic (laser) inputs were noisy (wide-band; Poisson). Furthermore, firing regularity determined the strength of the resonance with noisy extrinsic inputs, but not for regular inputs. These findings (difference Poisson vs. regular, and multiple resonant modes) suggest that the filtering exhibited by VTA dopamine neurons is not merely linear, but exhibits a non-linearity.

### 3.4. Neuron-Neuron Interactions Induced by Poisson Stimulation

**Figure 8:**
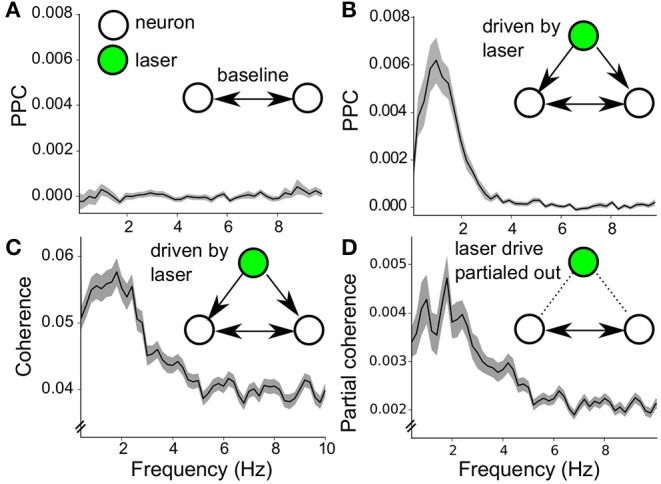
Noise induced synchrony during Poisson stimulation. **(A)** Baseline PPC spectrum (stimulation off) of pairwise neuron-neuron interactions (neuron pairs = 347) within the 7 recorded populations (mean ± sem). **(B)** Same calculation as in **(A)** during laser stimulation (mean ± sem). The peak around 1Hz indicates synchrony among pairs of VTA dopamine neurons (neuron pairs = 347). **(C)** Coherence spectrum of neuron-neuron interactions during stimulation on (mean ± sem, neuron pairs = 347), confirming the results in **(B)**. **(D)** Partial coherence spectrum (mind the y-axis, mean ± sem, neuron pairs = 347). Around 10% of the coherence in **(C)** is not directly related to the common drive, as it is taken into account during the coherence computation.

The dopamine neurons in the VTA population were able to respond collectively to Poisson stimulation, due to the latter's broadband frequency content (Figure 5C). Can such common noise stimulation induce a structured network state? To this end, the PPC was computed between all pairs of neurons within the population during stimulation off as well as stimulation on periods. The mean PPC spectrum during stimulation off (Figure 8A) showed no prominent PPC peak in the baseline pairwise neuron interactions, the values were never different from zero [Note that during baseline, only about 10–20% of neurons showed significant spike-spike synchronization, and only when the oscillation frequencies of the neurons match, van der Velden et al. (2019)]. During stimulation, the PPC spectrum has a large peak around 1 Hz (Figure 8B), which suggests neuron-neuron synchrony driven by the Poisson stimulation at selective frequencies.

The results on noise-induced-synchrony can be further refined because we have full knowledge of the timing of the Poisson laser pulses and thus can factor out its driving influence on the neuron-neuron synchrony. The component of the neuronal interactions due to common drive by the laser pulses was separated by computing the coherence and the partial coherence (see methods) (Rosenberg et al., 1989, 1998; Sun et al., 2004; Brett et al., 2009). The coherence (Figure 8C) corroborated the results of the PPC for neuron-neuron synchrony (Figure 8B). The partial coherence showed that about one-tenth of the coherence between pairs of neurons was not a direct result of the common drive by the laser pulses (Figure 8D, note the different scale of the y-axis). Thus the wide-band Poisson regime was capable of inducing a synchronous state across the VTA network, mainly due to common drive of the neurons (noise-induced-synchrony Galán et al., 2006; Ermentrout et al., 2008). Interestingly, part of the neuron-neuron interactions was not directly attributable to common drive, which points to emergent network properties during noise stimulation.

## 4. Discussion

VTA dopamine neuron exhibited spontaneous rhythmic firing with oscillation frequencies between 1 and 5 Hz in agreement with our earlier reports (van der Velden et al., 2017, 2019). Optogenetic stimulation using laser pulses affected the timing of the spikes. This spike timing modulation induced synchrony between the spikes and the laser pulses, which was used to study input resonance properties of the dopamine neurons and their network. VTA dopamine neurons resonated to the laser pulses in both the regular and the Poisson stimulation regime. The resonance spectra showed varying strength of phase locking between neuronal spikes and laser pulses related to the intrinsic oscillation frequency of the neurons. The VTA dopamine neurons had a different resonance response to Poisson than to regular input. The dominant resonance frequency to regular stimulation was almost 100 % higher than the one to Poisson stimulation. During regular stimulation the resonance frequencies were similar to the intrinsic oscillation frequency, as well as 0.5 and 2x this intrinsic oscillation frequency. For Poisson stimulation the peak resonance (at 1.5 Hz) scaled with the sub harmonic of the oscillation frequency (at 2.9 Hz). Additionally, for Poisson stimulation, the strength of the resonance also correlated with the intrinsic regularity of the auto oscillator. The separation of the resonance behavior into two frequency domains for regular vs. noise stimulation could mediate selective information exchange with different brain areas. Information could be processed independently between regular direct drive and noisy background signals, representing respectively local and long range communications. In all, this dynamic resonance behavior means that lateral VTA dopamine neurons behave as non-linear filters; at the same input frequencies they respond differently to narrow (regular) and broad-band (Poisson) input, through altered filter characteristics based on spike timing synchrony.

The broad-band nature of Poisson stimulation allowed us to drive simultaneously recorded neurons effectively, as each neuron can resonate to its preferred frequency. This common drive induced neuron-neuron synchrony (pairwise PPC), which was below the intrinsic oscillation frequency. Such noise-induced-synchrony has rarely been recorded in a neuronal system and is a recent addition to our understanding of the brains' function in the presence of noise (Galán et al., 2006; Ermentrout et al., 2008; Hata et al., 2010). Our study suggests that the lateral VTA with its weakly-coupled intrinsic oscillators and resonance characteristics is a suitable system for studying emergent network properties beyond the noise-induced-synchrony that is indicated by our results.

Fujisawa and Buzsáki (2011) hypothesized that the VTA is the pacemaker of low frequency local field rhythm (their data shows it between 1 and 5 Hz with a peak around 3 Hz), which entrains the prefrontal cortex and the hippocampus (Lisman and Grace, 2005; Fujisawa and Buzsáki, 2011; Kim et al., 2012). In our study the intrinsic oscillation frequencies of VTA dopamine neurons were between 1 and 5 Hz *in vitro*. The intrinsic oscillation frequency can be increased with extracellular glutamate (van der Velden et al., 2019). Under regular stimulation dopamine neurons resonate most strongly around 2.8 Hz, but also showed resonance at 2x the intrinsic oscillation frequency. Poisson stimulation induced noise-induced-synchrony among VTA dopamine neurons at 1.5 Hz. These results provide mechanisms underlying a pacemaker role for the VTA. Additionally, our data show that the lateral VTA has self organizing properties, as it exhibits neuron-neuron synchrony at selective frequencies while being driven by broad-band noise. The selective frequency during noisy stimulation is a 0.5x sub harmonic of its mean oscillation frequency. VTA dopamine neurons that fire with higher intrinsic regularity are able to resonate stronger at this sub harmonic. The VTA's self-organizing frequency-selective output during noise-like input thus represents a mechanism for low-frequency pacemaker activity.

We did not study the cellular mechanisms behind the sub oscillation frequency resonance to Poisson pulses, which likely involves sub-threshold oscillations (Lampl and Yarom, 1997) as described in several models of the dopamine neuron (Wilson and Callaway, 2000; Medvedev et al., 2003; Kuznetsov et al., 2006). We hypothesize that the observed resonance can be understood as the stochastic drive of a non-linear oscillator, which has a preferred stimulus phase within the spike cycle (i.e., halfway the spike cycle) (Tiesinga, 2002; Hata et al., 2010). Following this, Poisson stimuli are less likely to fall within the right time window each spike cycle, than every other cycle, every third or fourth cycle and thus will prefer to push the neuron with sub-harmonics of its intrinsic oscillation frequency.

Our findings have broad functional implications. The VTA has long been implicated in stimulus-reward coupling, with reward prediction based on an internal time code. However, the neural substrate of such a time code in the range of seconds is as yet unknown (Schultz, 1997; Lak et al., 2016). VTA dopamine neurons' phasic activity is proposed to act as a reward prediction signal, which is then modulated by GABAergic VTA neurons that receive external input (Cohen et al., 2012). Moghaddam et al. (2017) indeed showed that VTA network properties are critical to understanding the encoding of information about serial behavior. An underlying timing structure of the reward prediction is needed for such encoding and behavior. Our results suggest that the lateral VTA acts like a filter bank, whose neuron sub-populations resonate to different frequencies in the laser driven input. The neurons in these sub-populations are organized by having a similar or harmonically related intrinsic oscillation frequency. The output of these oscillating sub-populations encode the timing information in the input and could be used as a time code by down-stream target areas of the lateral VTA and as a reward prediction error code in general.

Clinically the VTA has been discovered as a target for deep brain stimulation (DBS) in the treatment of pharmacoresistant cluster headache (Akram et al., 2016, 2017). The frequency used for electrical stimulation of the VTA in this form of DBS therapy is 180 Hz, but no optimization has as yet been undertaken. This frequency is so high in comparison to the intrinsic VTA firing rates that if might best be considered as noisy stimulation. DBS is also a therapy of last resort in pharmacoresistant epilepsy, mostly at frequencies around 120 Hz, but here it could be demonstrated in an animal model that Poisson distributed stimulation was more effective than fixed frequency stimulation with the same energy (Wyckhuys et al., 2010).

Our research indicates that the VTA network is a non-linear filter bank when responding to external input, which can decode timing information related to stimulus-reward processing. These network states are self-organizing as they impose structure on noisy input and could underlie a pacemaker role of the VTA, linked to entrainment of the hippocampus and the prefrontal cortex during cognitive tasks.

## Data Availability Statement

The datasets generated for this study are available on request to the corresponding author.

## Ethics Statement

The animal study was reviewed and approved by Ethics Committee of the Swammerdam Institute for life sciences of the University of Amsterdam.

## Author Contributions

LV, MV, and WW designed the study, contributed to the data analysis, and wrote the manuscript. LV performed the experiments. LV and MV performed the data analysis.

### Conflict of Interest

The authors declare that the research was conducted in the absence of any commercial or financial relationships that could be construed as a potential conflict of interest.
